# Prioritizing Elective Surgery During the COVID-19 Pandemic Has Caused Age-Related Inequality: a Multicenter Study

**DOI:** 10.1007/s42399-021-01080-2

**Published:** 2022-01-10

**Authors:** Mikko Uimonen, Ilari Kuitunen, Ville Ponkilainen, Ville M. Mattila

**Affiliations:** 1 Central Finland Hospital Nova, Department of Surgery, Hoitajantie 3, 40620 Jyväskylä, Finland; 2 University of Eastern Finland, School of Medicine, Yliopistonranta 1, PL 1627, 70211 Kuopio, Finland; 3 Mikkeli Central Hospital, Department of Pediatrics, Porrassalmenkatu 35-37, 50100 Mikkeli, Finland; 4 Tampere University Hospital, Department of Orthopaedics, Elämänaukio, Kuntokatu 2, 33520 Tampere, Finland; 5 Tampere University, Faculty of Medicine and Health Technology, Arvo Ylpön katu 34, 33520 Tampere, Finland

**Keywords:** COVID-19, Epidemiology, Surgery, Elective, Public healthcare

## Abstract

The concern has been that this prioritization has resulted in age-related inequality between patients, with the older population suffering the most. The aim of this multicenter study was to examine the differences in incidence and waiting times of elective surgeries by age during the SARS-CoV-2 coronavirus disease (COVID-19) pandemic in Finland. Data on elective surgery (88 716 operations) were gathered from three Finnish public hospitals for the years 2017–2020. Surgery incidence and waiting times stratified by age groups (younger than 18, 18 to 49, 50 to 69, and 70 or older) were examined, and the year 2020 was compared to the reference years 2017–2019. The mean annual, monthly, and weekly waiting times were calculated with 95% confidence intervals (CI). The first COVID-19 wave decreased surgery incidence most prominently in patients younger than 18 (incidence rate ratio [IRR] 0.64, CI 0.60–0.68) and 70 or older (IRR 0.68, CI 0.66–0.70). After the first wave, the incidence increased in patients aged 50 to 69 and 70 or older by 22% and 29%, respectively. Among patients younger than 18, the incidence in 2020 was 15% lower. In patients younger than 18, waiting times were at mean of 43% longer in June to December compared to the reference years. In patients aged 18 to 49, 50 to 69, and 70 or older, waiting times increased in May but recovered to normal level during fall 2020. COVID-19 decreased the incidence of surgery and led to increased waiting times. Clearing of the treatment backlog started with older patients which resulted in prolonged waiting times among pediatric patients.

## Introduction

In March 2020, the spread of SARS-CoV-2 coronavirus disease (COVID-19) led to nationwide lockdown in Finland. To prepare for the pandemic, healthcare reorganizations were implemented, and non-urgent surgery was postponed. The lockdown was lifted in June, and elective surgeries were resumed. During the second COVID-19 wave in September 2020, cancellations were mainly avoided, and attempts were made to clear the cumulated backlog in elective surgery [1]. Due to limited resources, it was necessary to prioritize the clearing of the backlog to focus on the most urgent patients. Finland was able to prevent a major backlog in oncological surgeries and by careful planning the rate remained nearly unchanged during the pandemic [2]. However, the concern has been that this prioritization has resulted in age-related inequality between patients, with the older population suffering the most [3].

This multicenter study examined the differences in incidence and waiting times of elective surgeries by age during the COVID-19 pandemic in Finland.

## Methods

This study was performed in three large public hospitals in Finland (Central Finland Hospital, Mikkeli Central Hospital, and Tampere University Hospital) serving a catchment area of 900 000 people. Data on elective surgeries were gathered from hospital registers for the years 2017–2020. The patients were divided into four groups according to age: younger than 18, 18 to 49, 50 to 69, and 70 or older. In Finland, healthcare is publicly funded and accessible for all citizens. According to Finnish law, elective surgeries must be performed within 6 months from the initial decision for surgical treatment. Waiting times in days were calculated from the time interval between the date of the preoperative assessment in hospital outpatient clinics and the date of the operation. As the focus was on elective surgery, only procedures with waiting times over 14 days were included.

Age group stratified monthly and annual elective surgery incidences with 95% confidence intervals (CI) were calculated for the year 2020 and commonly for the reference years 2017–2019 by Poisson exact method. Population data were obtained from the Statistics Finland open database [4]. Incidence rate ratios (IRR) were calculated between 2020 and the reference years. The monthly and annual mean waiting times with 95% CIs were examined and compared between 2020 and the reference years. The comparison was stratified by age groups. Statistical analysis was performed using R (4.0.3) statistical software. Ethical committee approval was not required due to the retrospective register-based study design [5]. This report has been reported according to STROBE guideline.

## Results

A total of 88 716 elective operations were performed in the participating hospitals during 2017–2020 (21 979 in Central Finland Hospital, 9 315 in Mikkeli Central Hospital, and 57 422 in Tampere University Hospital). Of all operations, 46 306 (52%) were performed for female patients and the mean age of the patients was 56.4 (SD 19.9).

**Fig. 1:**
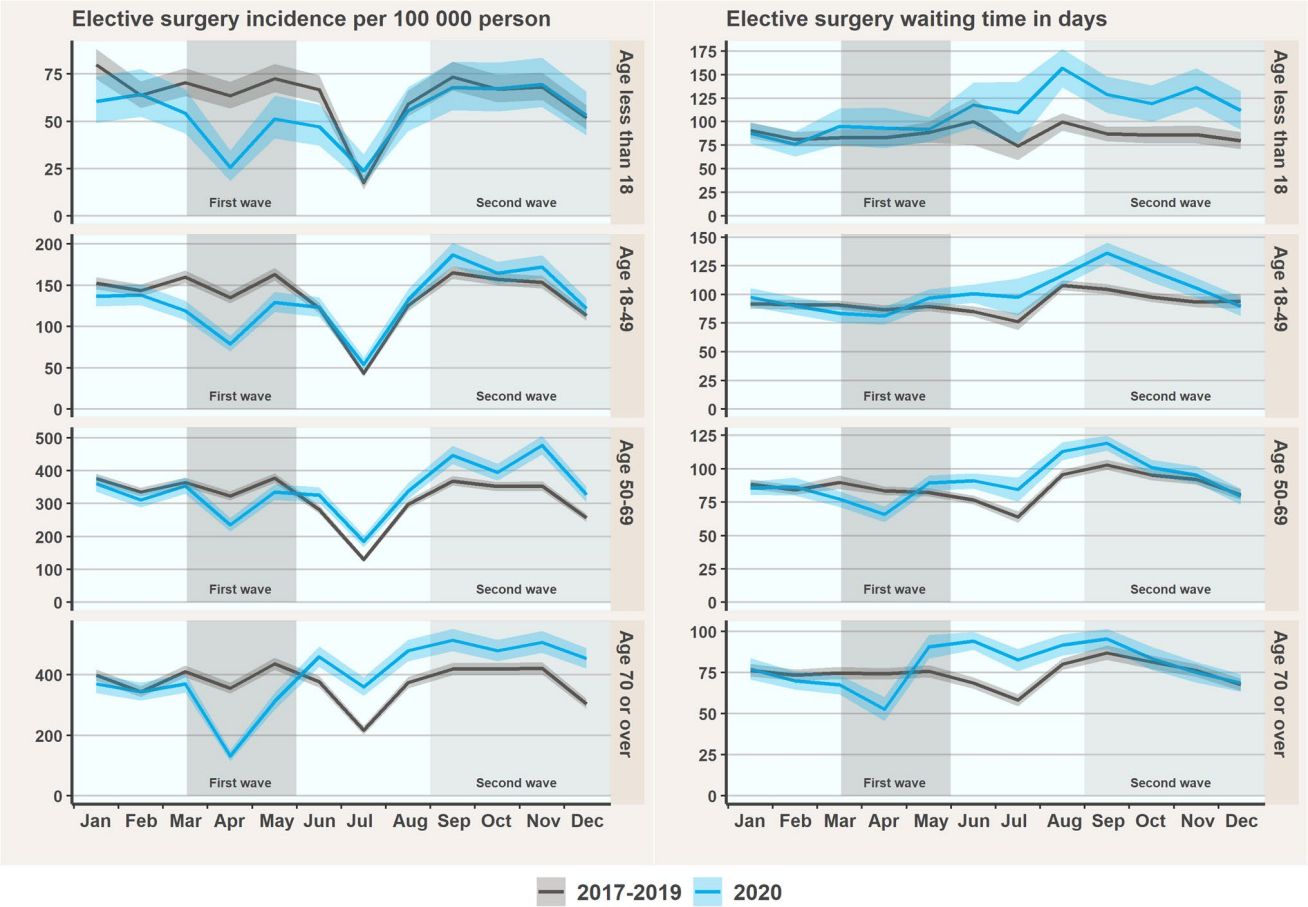
Monthly incidences and mean waiting times in elective surgery with 95% confidence intervals by age group in 2020 and the reference years

The first wave of the pandemic during March to May 2020 decreased incidence of surgery in all age groups, with the most prominent decreases in patients younger than 18 (IRR 0.64, CI 0.60–0.68) and 70 or older (IRR 0.68, CI 0.66–0.70; Fig. 1). After the first wave, surgery incidence recovered and followed the reference level until the end of the year, resulting in a 15% decrease in total annual incidence among patients younger than 18 (annual IRR 0.85, CI 0.82–0.88). In the patients aged 50 to 69 and 70 or older, rebound effects were observed after the lockdown from June to December, with increases in incidences of 22% and 29%, respectively. Among patients aged 18 to 49, a slight rebound effect was observed later in September and thereafter.

The mean waiting time for elective surgery during the years 2017–2020 was 88 days. In patients younger than 18, waiting times remained unchanged during the first COVID-19 wave from March to May 2020, although they were considerably longer after the first wave without recovering by the end of 2020 (Fig. 1). Moreover, from June to December, the overall increase in waiting times was 43% among patients younger than 18. At the beginning of the first wave, waiting times in patients aged 50 to 69 and 70 or older occasionally decreased but increased above the reference levels in May in both groups. Waiting times recovered in October in patients aged 50 to 69 and 70 or older, with an average increase of 17% and 21% between May and September, respectively. In patients aged 18 to 49, the recovery in waiting times occurred later in December. On average, waiting times in patients aged 18 to 49 were 19% longer in May to November.

## Discussion

The lockdown decreased the incidence of elective surgery in all patient groups, leading to a subsequent increase in waiting times after the lockdown. However, the prioritizing of older patients after the lockdown resulted in a faster recovery in waiting times when compared to younger patient groups.

The most prominent influence of the lockdown on the incidence of elective surgery was observed in pediatric and older patients. Although severe COVID-19 disease has been shown rare among children [6–8], healthcare policies at the beginning of the pandemic were likely based on the lowest risk principle with limited research-based knowledge. With prior knowledge of respiratory diseases, the fear was that children and the older population would suffer the highest disease burden [9, 10]. Hence, our findings probably reflect obscurity and concerns about the risk for infection and severe COVID-19 disease among these patient groups at the beginning of the pandemic. After the lockdown, the surgery incidence increased substantially among patients aged 70 and older as well as in patients aged 50 to 69. Similarly, the recovery in waiting times started with the oldest patients. Nevertheless, in patients younger than 18, waiting times were considerably longer after the lockdown (43% increase) without recovering by the end of 2020. This finding may reflect the low risk related to the postponement of surgery in children. Similar findings on surgery postponements in the pediatric population have been previously reported [11, 12].

Contradictory to previous concerns, the findings of this study show that the prioritizing of surgery was actually performed in favor of the older population, not against them [3]. It seems, however, that the most unfavorable influence of the prioritizing of surgery has fallen on pediatric patients. Along with the literature showing that lockdown restrictions have especially endangered the mental health and well-being of the younger population [13], our findings suggest that this disparity also extends to the field of healthcare.

The main strength of this study is the representative data from three large public Finnish hospitals with reference data from three previous years. The main limitation of this study is the shortcomings of the register-based data, which eliminate the possibility to estimate the influence of prolonged waiting times on surgery outcomes or disease severity and prioritizing for individual patients.

## Conclusions

In conclusion, the healthcare lockdown at the onset of the COVID-19 pandemic decreased the incidence of surgery and led to increased waiting times. Clearing of the treatment backlog after the lockdown started with older patients and resulted in a faster recovery in waiting times for older patients. The most unfavorable influence of the prioritizing of surgery fell among pediatric patients.

## Data Availability

Available upon reasonable request from the corresponding author.

## Code Availability

Available upon reasonable request from the corresponding author.
